# Long Term Persistence of IgE Anti-Varicella Zoster Virus in Pediatric and Adult Serum Post Chicken Pox Infection and after Vaccination with Varicella Virus Vaccine

**Published:** 2009-12

**Authors:** Tamar A. Smith-Norowitz, Joby Josekutty, Jonathan I. Silverberg, Hadar Lev-Tov, Yitzchok M. Norowitz, Stephan Kohlhoff, Maja Nowakowski, Helen G. Durkin, Martin H. Bluth

**Affiliations:** 1 *Departments of Pediatrics, Center for Allergy and Asthma Research, S.U.N.Y. Downstate Medical Center, Brooklyn, New York, USA;*; 2 *Department of Pathology, Center for Allergy and Asthma Research, S.U.N.Y. Downstate Medical Center, Brooklyn, New York, USA;*; 3 *Department of Medicine, Center for Allergy and Asthma Research, S.U.N.Y. Downstate Medical Center, Brooklyn, New York, USA;*; 4 *Department of Pathology, Wayne State University School of Medicine, Detroit, Michigan, USA*

**Keywords:** IgE, varicella zoster virus, varicella zoster virus vaccine

## Abstract

The production of IgE specific to different viruses (HIV-1, Parvovirus B19, RSV), and the ability for IgE anti-HIV-1 to suppress HIV-1 production *in vitro*, strongly suggest an important role for IgE and/or anti viral specific IgE in viral pathogenesis. Previous studies in our laboratory were the first to report the presence of IgE anti-varicella zoster virus (VZV) in an adolescent patient with shingles. However, the presence and long term persistence of IgE anti VZV antibodies has not been studied in adults. The presence of serum IgE in addition to IgE and IgG anti-VZV antibody in sera were studied in children (N=12) (0–16 y/o) and adults (N=9) (32–76 y/o) with either a past history of (wild type) chicken pox (N=7 children, 9 adults) or 5 years after vaccination with varicella zoster (N=2 children) (Varicella virus vaccine live, Oka/Merck), as well as in non-infected subjects (N=3 children). Of the patients who had a positive history of chicken pox 13 of 16 (81%) contained IgE anti-VZV antibodies; they were both serum IgE_Hi_ (>100 IU/ml) and IgE_Lo_ (<100 IU/ml). Of the patients who were vaccinated, IgE anti-VZV antibodies were undetected. In contrast, serum from the patients without a history of chicken pox or vaccination did not make either IgE or IgG anti-VZV antibodies. This is the first demonstration of the existence of IgE anti-VZV antibodies, and its long-term persistence in serum of previously infected subjects. Future studies regarding the functional role of anti-viral IgE and its relationship to VZV are warranted.

## INTRODUCTION

Studies in our laboratory have previously demonstrated that IgE may play an important role in immunity to specific viruses, including Parvovirus B19 (1), HIV-1 (2, 3), and VZV in an adolescent patient with shingles (4, 5). Other studies in our laboratory also identified IgE anti- spirochete antibodies (*B. Burgdorferi*) and its persistence one year later in serum of lyme-infected children (6).

Studies of others have identified IgE anti-virus antibodies in several viral infections including respiratory syncytial virus (RSV) (7, 8), parainfluenza (9), HTLV-1 (10), Puumala virus (11), HSV-1, HSV-2, and Epstein-Barr virus (12). The presence of serum antibody IgG to VZV and its correlation with immunity to varicella has been shown (13, 14); however, there have been no reports of the presence or the antigenic specificity of IgE anti-VZV in pediatric or adult patients with a past history of chicken pox infection or vaccination against VZV.

This is the first description of the presence of IgE anti-varicella zoster virus antibodies and its long term persistence in serum of previously infected adults or pediatric subjects. The exact role of IgE in VZV infection remains unknown; however, the presence of IgE anti VZV antibodies in infection 10–40+ years after chicken pox infection, warrants further investigation of the biological significance, if any, of these antibodies.

## MATERIALS AND METHODS

### Patient specimen description

Peripheral blood (3 ml total) was obtained from both pediatric (N=12) (0–16 yrs old) and adult (N=9) (m/f, 32–76 yrs old) subjects from the SUNY Downstate Allergy Clinic, who were atopic and non atopic. Atopic subjects were skin prick positive (N=4) for environmental (e.g. mixed tree and grass, ragweed, weeds, and dust mite) or food allergens. Exclusion criteria included patients with milk allergies. At the time of study, the subjects had not received allergy therapy, and were not being treated with any medication. Subjects did not have a past history of parasite infection. Approval was obtained from the SUNY Downstate Institutional Review Board, and the procedures followed were in accordance with institutional guidelines involving human subjects.

Subjects were interviewed about past history of wild type chicken pox infection (WT) or whether they had been vaccinated (Varicella virus vaccine live, Oka/Merck). All adults had wild type varicella infection prior to the advent of the Varicella virus vaccine (1997). Time post infection for chicken pox infection in children was 10–14 years; in adults 15–40 yrs. Past history of wild type chicken pox infection was confirmed by positive titers for IgG varicella zoster virus by Quest Diagnostics Inc. (Teterboro, NJ).

### Blood specimens/Immunoglobulin Determinations

For studies of serum immunoglobulins (Ig), blood was collected into red top monoject tubes (Sherwood Medical, St. Louis, MO) and sent to Quest Disagnostics, Inc. for Ig determinations.

Total serum immunoglobulin E (IgE) was determined by The Clinical Immunology Laboratory at SUNY Downstate (Brooklyn, NY) according to standard procedure (UniCAP Total IgE Fluoroenzymeimmunoassay). Reference range for healthy adult or child serum: IgE: 20–100 IU/mL.

### Varicella Zoster Virus serum antibody detection: EIA

**IgG.** Serum IgG to varicella zoster virus were determined by enzyme immunoassay (EIA) performed by Quest Diagnostics Inc. (Teterboro, NJ) according to standard procedure. Data are reported as ratio report. (Ranges for VZV Ab IgG: Negative <0.91, Positive: >1.09).

### Varicella Zoster Virus serum antibody detection: Immunoblot

**IgE.** The presence of IgE anti-varicella zoster virus antibodies was determined by immunoblot (dot blot), as previously described (5). Briefly, purified Varicella Zoster Virus antigen (Fitzgerald Industries International, Inc., Concord, MA; (Strain VZ-10, Cat# 30-AV09) (5ul) (1.46 mg/ml protein conc., diluted 1:100 in diluent) was pipetted onto nitrocellulose membrane strips (BIO-RAD Laboratories, Hercules, CA) and let dry. Nitrocellulose membrane was then soaked in a 5% milk powder (Immunetics Inc., Boston, MA) solution (Tween 20 (0.05% Tween20 (Sigma) in tris buffered saline (20mM Tris-HCL (Sigma), 150 mM NaCl, pH 7.5 (Sigma). Nitrocellulose membranes were then incubated with serum samples (100 ul) (diluted in 2 ml TBS-Tween 20) for 1 hr at room temperature, after which goat polyclonal anti-human IgE (ICN), diluted 1:100 in TBS-Tween 20 and 1% milk in TBS-Tween 20 (1 ml), was added to membranes, and incubated for 1 hr on a shaker at room temperature. The membranes were then washed three times with TBS-Tween 20. Nitrocellulose membranes were then incubated with rabbit anti-goat peroxidase labeled antibody (ICN), diluted 1:2000 in TBS-Tween 20 and 1% Milk for 1 hour on a shaker. The membranes were then washed 3 times with TBS-Tween 20, and then developed in TMB substrate solution (2 ml). The membranes were then removed from the TMB substrate solution, at which time they were read dried, and scanned (Gel Doc 2000 System with specific The Discovery Series: Quantity One software BioRad, Hercules, CA). Relative IgE levels were quantified by using NIH ImageJ software. In order to eliminate effects of non-specific labeling, for each immunoblot, the color reaction of the portion of nitrocellulose without varicella antigen was considered as background signal, and was subtracted from the portion of nitrocellulose with varicella antigen. Controls consisted of blots performed in the absence of Varicella Zoster Antigen which were negative in all cases tested (data not shown) and for IgE anti-HIV-1 abs, which were also negative in all cases tested (data not shown). Data are reported as fold change which is defined as the difference between the amount of signal obtained from the color in the sample, compared with the white background signal.

### Statistical analysis and correlations

In order to demonstrate the specificity of our IgE anti-VZV assay, we performed correlations between levels of specific IgE anti-VZV and total serum IgE levels and background signal from the portion of nitrocellulose without varicella antigen. Pearson’s correlation coefficients and the corresponding p-values were calculated to test the hypothesis that IgE anti-VZV positivity is specific. Significance between groups were determined by ANOVA (p<0.05).

## RESULTS

### Characteristics of Study Subjects

Serum IgE levels and IgE and IgG anti-varicella zoster virus were studied in children (N=12) (m/f 0–16 y/o) and adults (N=9) (32–76 y/o) with either a past history of (wild type) chicken pox (N=7 children, 9 adults) or 5 years after vaccination with varicella zoster (N=2 children) (Varicella virus vaccine live, Oka/Merck), as well as non-infected children (N=3). (UniCAP total IgE Fluoroenzymeimmunoassay, EIA, Western blot) (Table 1). One of our vaccinated subjects was removed due to exclusion criteria (see methods). Subjects with a past history of chicken pox infection, or who were vaccinated tested positive (>1.09, ratio report) for IgG anti-VZV abs (Table 1).

**Table 1:** Characteristics of study subjects and serum IgE levels

Patient	Sex/Age (years)	Appoximate Age of VZV onset (yrs)	Form of Varicella Inoculation	Serum IgE levels (IU/ml)	Serum IgG anti-VZV levels (+/−) b

1	F (13)	5	Wild – type	150^a^	+
2	M (10)	N/A	Vaccine – type (recombinant)	369^a^	+
3	F (9)	N/A	Vaccine – type (recombinant)	31	+
4	M (8)	4	Wild – type	256^a^	+
5	M (16)	3	Wild – type	52	+
6	F (14)	13	Wild – type	36	+
7	M (3)	9 mo	Wild – type	34	+
8	M (12)	3	Wild – type	80	+
9	F (13)	<10	Wild – type	163	+
10	M (0)	N/A	No history	2.3	−
11	M (47)	<10	Wild – type	346^a^	+
12	M (51)	<10	Wild – type	195	+
13	F (32)	11	Wild – type	15	+
14	F (49)	4	Wild – type	34	+
15	F (60)	40	Wild – type	30	+
16	M (37)	10	Wild – type	110	+
17	M (39)	22	Wild – type	150	+
18	M (35)	<10	Wild – type	241^a^	+
19	M (76)	<10	Wild – type	33	+
20	F (3)	N/A	No history	107	−
21	F (1)	N/A	No history	22	−

Patients with a past history of varicella zoster infection (wild-type), vaccination (recombinant), or no infection.

a Patient skin test (skin prick) positive for food or environmental allergens. Reference range for healthy adult or child serum: IgE: 20–100 IU/mL. N/A: not applicable.

b +/−: Ranges for VZV Ab IgG: Positive: >1.09, Negative <0.91, expressed as ratio report.

### Total IgE

Of those with a positive history of chicken pox, eight (4 children, 4 adults) had reduced serum IgE levels (<100 IU/ml); and eight (3 children, 5 adults) had elevated serum IgE (110–346 IU/ml). Vaccinated children, and children with no history of chicken pox had serum IgE levels which were low (Table 1).

### Anti-VZV Abs

**IgG.** Serum obtained from subjects who had wild type chicken pox or who were vaccinated had positive IgG anti VZV antibody levels (Antibody Index IgG: >1.09). In contrast, serum from non-infected (control) subjects did not make IgG anti-VZV antibodies (data not shown).

**Figure 1:**
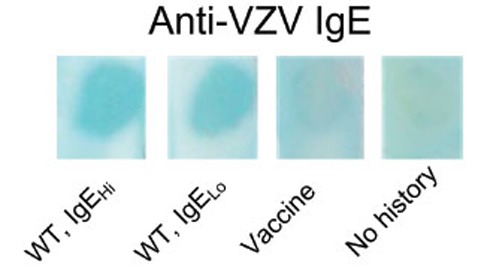
Western blot analysis of anti-varicella zoster virus (VZV) antibodies. Serum from subjects with either past history of VZV infection, vaccination or no infection was incubated with nitrocellulose strips containing VZV antigen (see Materials and Methods). Lane 1: representative blot of subjects with either past history of VZV who have elevated (>100 IU/ml) IgE levels. Lane 2: representative blot of subjects with either past history of VZV who have low (<100 IU/ml) IgE levels. Lane 3: representative blot of VZV vaccinated subject. Lane 4: control subjects, no history of infection.

**Figure 2:**
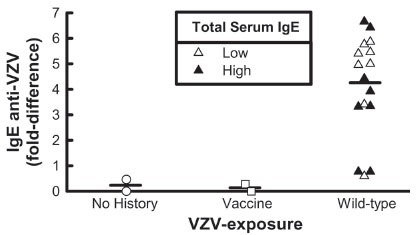
Quantitative analysis of IgE anti-VZV. IgE anti-VZV antibodies were determined in patients who have high (black triangles) or low (white triangles) IgE levels and had either no history of VZV exposure (white circles) or were exposed to VZV vaccine (white squares). Data are expressed as fold difference of IgE-anti-VZV antibody (defined as the difference between the amount of signal obtained from the color in the sample, compared with the white background signal). Significance was observed for “Wild-type” when compared with “No History”/“Vaccine” groups (p=0.04).

**IgE.** Serum obtained from subjects who had wild type chicken pox 13 of 16 (81%) had positive dot blots for IgE anti VZV antibodies; they had high and low levels of serum IgE (Fig 1, lanes 1, 2). In vaccinated (serum IgE negative), IgE anti varicella zoster virus antibodies were undetected (Fig 1, lane 3). In contrast, serum from the children without a history of chicken pox or vaccination did not contain IgE anti-VZV antibodies (Fig 1, lane 4); they had low levels of serum IgE. No correlation was found between the presence of IgE anti-VZV and total serum IgE levels (p= 0.62) or background signal (p= 0.81) (Fig 2).

## DISCUSSION

This is the first study demonstrating the presence of IgE anti-VZV antibodies in both children and adults with a past history of chicken pox infection (10–40 years +). VZV is a member of the Herpesviridae family, a group of large DNA viruses that replicate in the cytoplasm of virus-infected cells (15). Chicken pox is a highly contagious disease caused by VZV, spread through respiratory droplets and/or direct contact with vesicles (16). It is usually mild, but can be serious, especially in young infants and adults. Laboratory diagnosis includes either the VZV polymerase chain reaction (PCR) as well as the direct detection of virus in cell cultures, or the detection of specific VZV immunoglobulins of classes IgG, IgM and IgA (ELISA) (17). Thus, clinical diagnosis is mostly supported by serologic findings (18). In 81% of our WT chicken pox infected patients with both low and high serum IgE levels, and not in our vaccinated patient, IgE anti-VZV antibodies were detected by immunoblot and confirmed using semi quantitative analysis (Fig 2). One of the most remarkable findings presented here is the long-term persistence of these antibodies (10–40+ years post chicken pox infection). However, total serum IgE levels were variable and no correlation was found between the presence of IgE anti-VZV and total serum IgE levels. These results suggest that there may be a specific component in chicken pox infection which stimulates the production of IgE anti-VZV antibodies which is not contingent or correlated with total serum IgE levels and that these responses are long lived. Further, the subjects with a history of WT infection might have had an “induction phase” early on to the antigen, which the vaccinated subjects never experienced, and therefore when they have persistent exposure to the wild type VZV antigens, continue to make antibodies against it. Studies of Pellegrino, *et al*. (3) have shown that HIV-1 specific IgE persists after 210 days and was also able to suppress HIV-1 production *in vitro* (3). Further, glycoprotein I (gI) of VZV has been shown to contribute to viral virulence, and is an important target for T cell control of viral replication. It has been reported that many decades after infection, relatively high frequencies of gI-specific interferon gamma were detected ex-vivo and are dominated by CD4+ T cells (19). Earlier studies in our laboratory have demonstrated that the levels of IgE specific for an antigen can be critical for an IgE mediated effect irrespective of total serum IgE levels (20, 21). However, we are aware of the limitations of our study in that immunoblots only demonstrate the presence and specificity of antibodies and cannot provide quantitative information of the level of antigen specific serum IgE antibodies; therefore semi-quantitation of the blot was performed (Fig 2). It could be that the levels of antigen specific IgE are more critical than total IgE levels in facilitating an antiviral response as we have shown for HIV-1 (21) and other diseases (20). Although the role of IgE anti-VZV antibodies are unknown, it could be that IgE could serve an immunomodulatory function. As such the propagation of IgE may be different in vaccinated vs wild type infection as a result of differences in disease pathophysiology. Our previous demonstrations of differences in the presence and persistence of IgE anti-viral antibody generation in viral infection (HIV, Parvovirus) and anti-viral effects (viral inhibition, cytotoxicity) (1–3, 21) may be in play in VZV infection as well. Further studies will determine the functional relationship of IgE anti-VZV antibodies in VZV.

VZV vaccination has been recommended by the Center for Disease Control (CDC) for all health care workers (22) as well as children. Patients are given two doses of the live attenuated varicella vaccine (Varivax©, Merck and Company, Inc, West Point, PA) according to the recommended schedule of the American Academy of Pediatrics (23). Studies have reported the presence of IgG antibodies to VZV in healthy (non-vaccinated) children (24, 25) and antibodies against VZV and rubella virus (26), in the absence of vaccination. VZV seroprevalence was highest in these children at 48 and 60 months old (25).

Future studies are necessary to elucidate the role of IgE in VZV infection and to determine whether IgE possesses any functional or immunomodulatory role in this disease, and whether it might have use as a potential biomarker for chicken pox infection.
